# Challenging the status quo: a framework for mechanistic and human-relevant cardiovascular safety screening

**DOI:** 10.3389/ftox.2024.1352783

**Published:** 2024-03-25

**Authors:** Brian Berridge, Jennifer Pierson, Syril Pettit, Norman Stockbridge

**Affiliations:** ^1^ B2 Pathology Solutions, LLC., Durham, NC, United States; ^2^ Health and Environmental Sciences Institute, Washington, DC, United States; ^3^ US Food and Drug Administration, Center for Drug Evaluation and Research, Silver Spring, MD, United States

**Keywords:** cardiac NAM, cardiovascular toxicity, cardiac toxicity, cardiac drug development, cardiac

## Abstract

Traditional approaches to preclinical drug safety assessment have generally protected human patients from unintended adverse effects. However, these assessments typically occur too late to make changes in the formulation or in phase 1 and beyond, are highly dependent on animal studies and have the potential to lead to the termination of useful drugs due to liabilities in animals that are not applicable in patients. Collectively, these elements come at great detriment to both patients and the drug development sector. This phenomenon is particularly problematic in the area of cardiovascular safety assessment where preclinical attrition is high. We believe that a more efficient and translational approach can be defined. A multi-tiered assessment that leverages our understanding of human cardiovascular biology, applies human cell-based *in vitro* characterizations of cardiovascular responses to insult, and incorporates computational models of pharmacokinetic relationships would enable earlier and more translational identification of human-relevant liabilities. While this will take time to develop, the ultimate goal would be to implement such assays both in the lead selection phase as well as through regulatory phases.

## 1 Introduction

Traditional approaches to preclinical drug safety assessment have supported an accepted level of protection for human patients from unintended adverse effects in clinical trials and post-marketing clinical use. These approaches are mostly based on animal studies and occur late in preclinical development as Investigational New Drug (IND)-enabling studies. These studies frequently reveal putative safety liabilities that can lead to attrition and ultimate termination in the drug development process (Laverty et al., 2011). There are a number of key challenges inherent in our current animal-based approach including its application late in development, lack of scalability for use in earlier stages of development, varying predictivity for human outcomes and little substantive mechanistic insight from these very morphology-based studies. Significant investment in time, people and fiscal resources is lost when attrition occurs at a late stage of development when a novel drug candidate is either terminated for safety concerns prior to clinical testing or, worse, in the clinical phase of development, a detriment to both product developer and patient. Even worse, that resource was not expended on an asset that could have been successful. In limited, but sometimes highly visible cases, safety liabilities occur in patients that were not detected in these preclinical studies (Type II or false negative errors) suggesting that traditional lead compound assessment approaches lack predictivity for some human outcomes (Laverty et al., 2011; Ferri et al., 2013; Waring et al., 2015). What is less quantifiable but equally detrimental to the goal of developing safe and effective medicines is attrition resulting from Type 1 or false positive errors, particularly findings identified in preclinical animal studies that are qualitatively or quantitatively irrelevant to patients (i.e., they are liabilities for animals but not humans) (Dogterom et al., 1992; Monticello et al., 2017). Accordingly, there are two fundamental challenges in contemporary preclinical drug safety assessment: development-limiting safety liabilities are not identified until late in nonclinical development (i.e., candidate selection and beyond) and safety-related attrition due to liabilities that are not relevant to humans represent lost opportunities for patients and drug developers.

Cardiovascular safety liabilities are well recognized to be significant contributors to the overall safety-related attrition in both preclinical and clinical studies (Laverty et al., 2011). Like most liabilities, they are most often identified in late preclinical or early clinical development where safety assessments are concentrated in the typical drug development scheme. Though some molecular mediators of the cardiovascular system are components of typical secondary (off-target) pharmacology screening and *in vitro* assessment for proarrhythmic hERG-binding is common, much of the assessment of drug-induced effects on the cardiovascular system relies on subjective and unblinded interpretation of samples from whole animal studies (Laverty et al., 2011; Bowes et al., 2012; Whitebread et al., 2016).

For cardiovascular risk assessment, single dose, non-rodent cardiovascular safety pharmacology studies are used to assess acute functional effects (Pugsley et al., 2010). Repeat-dose general toxicity studies in rodents and non-rodents characterize structural or morphological changes with histopathology, clinical chemistry, and hematology. For the most part, any functional consequences or causes of structural change are left to speculation since assessments of function are not common in repeat-dose animal studies aside from sporadic monitoring of the ECG in non-rodent studies (Berridge et al., 2013).

The current animal-based safety assessment approach attempts to mitigate the challenges of cross-species translation where international guidances recommend a 2-animal species safety assessment package (ICH Harmonised Tripartite Guideline, 2009). In this paradigm, a liability in either species is considered potentially human-relevant unless convincing mechanistic evidence can be developed to prove otherwise. This approach contributes to the risk of false positives, and, because it is inherently low-throughput and resource consumptive, it generally prevents the advancement of multiple candidates concurrently. It also poorly informs the mechanistic initiators of the injuries observed or their human relevance without additional time-consuming investigation.

The mechanistic insights gained through an evolved paradigm could help to proactively inform drug target relatedness and patient relevance as well as support the design of more sensitive clinical biomarker strategies. For example, when an unexpected liability is identified currently, an initial concern is whether that liability has any relation to the pharmacologic target of interest (as the development implications of this assessment are profound). Current phenotypic approaches offer no real opportunity to advance this understanding, often prompting additional investigation that consumes valuable patent life of the novel drug candidate. It is also difficult to predict the potential for a CV bioactivity to be a unique liability in a patient with pre-existing disease without some fundamental understanding of the mechanism of that bioactivity. For example, a drug that unintentionally activates or agonizes β-adrenergic receptors even at physiological levels may be a liability in a patient with heart failure where the β-adrenergic system is often upregulated and the patient is taking β-blockers. Likewise, drugs that inhibit phosphodiesterases that could induce vasodilation might be contraindicated in a patient on hypotensive therapy (Kloner et al., 2018; Schurtz et al., 2023.) Without sufficient insight into the mechanisms of these toxicities, our ability to stratify patient risk is hindered.

Safety assessment is increasingly challenging in a world where patients are experiencing more chronically progressive disease managed with long-term drug therapy. Assessment of acute safety liabilities or toxicity is profoundly easier to do than characterizing long-term liabilities of drugs in patients (particularly those with comorbidities). While animal-based safety assessment models can temporally replicate moderately long-term dosing (usually in the absence of comorbidities), a phenotypic outcomes-based paradigm reliant on animal studies provides little insight into the early subclinical biological activities of the drug that should be the target of mitigating the potential for harm. The addition of better insights into the human-relevance of the drug’s mechanism of action could better enable effective monitoring and assessment of a drug’s bioactivity in anticipation of potential clinical liabilities. As an example, clinicians currently use insights into modes of actions of drugs to manage these challenges in individual patients. The ability to manage and mitigate putative liabilities is informed by a better understanding of the bioactivity of the drug—insights that rarely come from describing biochemical and morphological outcomes in animal studies. The paradigm proposed here offers opportunities to systematically enhance our mechanistic understanding of pharmaceutical bioactivity.

**FIGURE 1:**
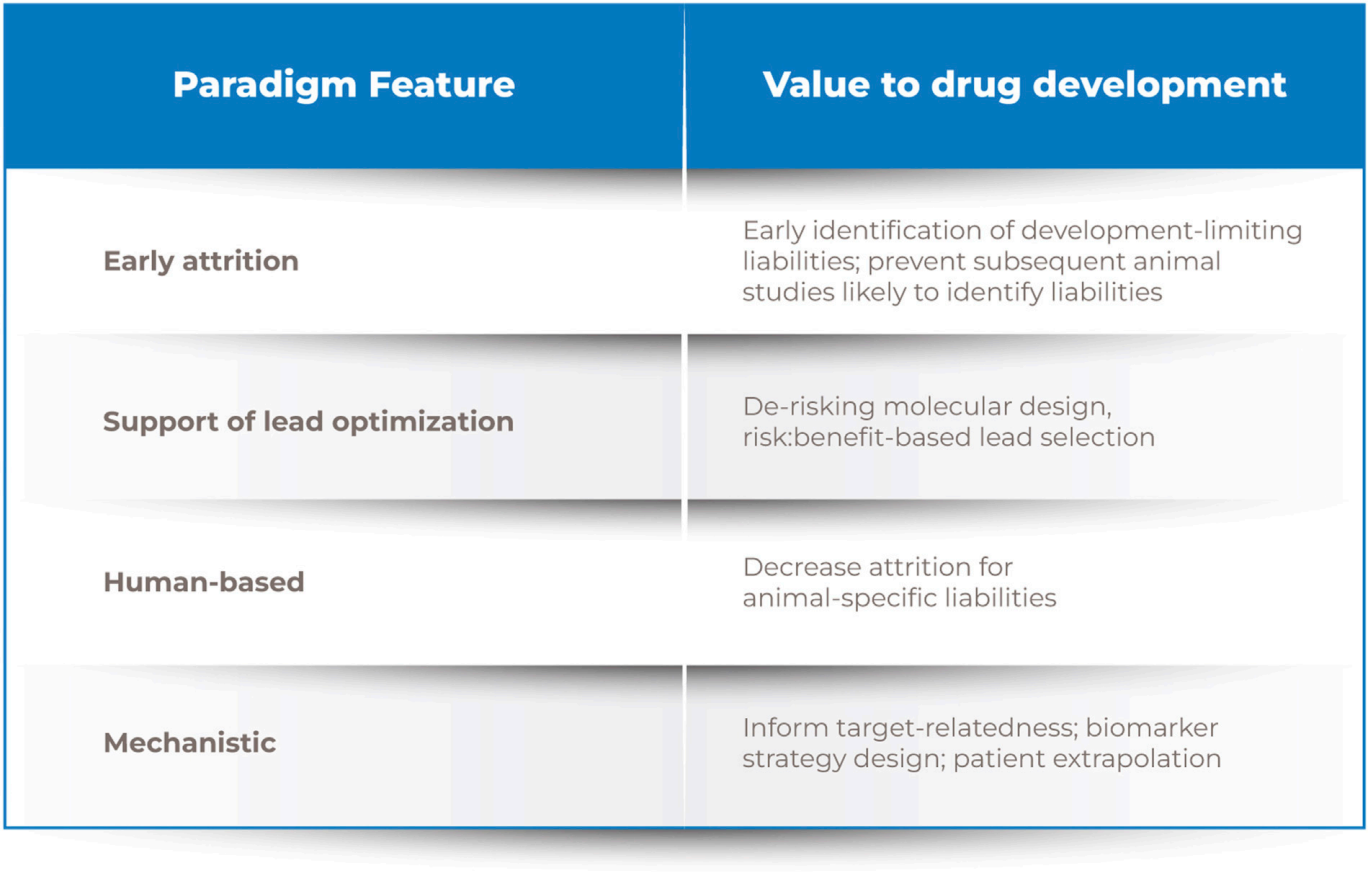
Drug discovery process. Note. A novel human-relevant and mechanistic screening paradigm positioned earlier in development would enable evidence-based lead selection and more informed preclinical and clinical safety assessments.

The authors propose that the development and implementation of a multi-tiered, human-relevant and mechanistic assessment that leverages our vast experience and understanding of how the human cardiovascular system responds to physiological or pathophysiological perturbation could meaningfully address limitations in the current paradigm and improve patient benefit, while also enhancing overall efficiency and reduce animal use. Unlike the current approach where critical assessments are left until the eve of clinical progression, a novel paradigm could be more objective and hypothesis-driven and supported by a progressive generation of mechanistically-informative data that enables safety assessment along a larger continuum of drug development decision-making (Figure 1). This approach will employ the use of new approach methodologies (NAM), defined as any technology, methodology, approach, or combination that can provide information hazard and risk assessment without the use of animals (*in silico*, *in chemico*, *in vitro*, *ex vivo*) (Stucki et al., 2022).

Our current CV safety assessment approach is potentially overly conservative, highly animal dependent, and often deficient in mechanistic insights. The proposal that follows describes a path to leverage our contemporary understanding of human biology, cardiovascular toxicity, and technical capabilities to enable a more useful approach that is more broadly applicable along the development continuum.

## 2 A new approach

Early drug discovery and lead optimization are often biased toward optimizing pharmacological potency and bioavailability to ensure patient benefit, yet that candidate will not become a marketed drug without an acceptable risk/benefit profile. Our re-envisioned approach seeks to shift safety considerations to earlier in the development paradigm. By incorporating defined assessments of bioactivity on key targets of CV relevance and responses to injury in simpler and higher-throughput modeling systems earlier than current safety or secondary pharmacology assessments, it may be possible to more expeditiously inform risk/benefit assessment, molecular design, and/or subsequent human studies (Figure 1). This leftward shift in the timeline for assessment is intended to enable more proactive identification and mitigation of human CV safety liabilities (Pierson et al., 2013).

The feasibility of defining and implementing such an approach is supported by our fundamental understanding of how the CV system works, knowledge of key pathways that have been implicated in adverse effects on the structure and function of the CV system, and data on the manifestations of drug-induced toxicity gained through our decades of experience modeling drug-induced CV toxicity (Haschek et al., 2013). Significant advances in scalable human-derived cell systems that model more *in vivo* and physiologically-relevant phenotypes, the ability to interrogate cell biology or pathobiology at increasingly mechanistic and molecular levels, and computational methods that quantitatively link *in vitro* exposures to *in vivo* exposures (IVIVE) can augment these historical datasets and provide additional insights into the human fidelity of the responses (Dick et al., 2010; Doherty et al., 2013; Sirenko et al., 2017; Bell et al., 2018; Grimm et al., 2018; Wambaugh et al., 2018; Gintant et al., 2019; Honda et al., 2019).

### 2.1 Leveraging our knowledge and experience–the “failure mode” concept

We have been evaluating the CV system for health, disease and toxicity in animals and human patients for centuries. We know its structural and functional constituents. We know its physiology, its pathophysiology and what it looks like when toxicity and disease manifests (Katz, 2010; Haschek et al., 2013.) The breadth of that understanding is an opportunity to invent a novel approach to evaluating drug effects that may be liabilities for patients. Though we continue to learn more, many of the molecular and mechanistic mediators of cardiac and vascular physiology are known. The heart rhythmically contracts to circulate blood throughout the body. Cardiac valves ensure unidirectional flow. Vascular arteries, capillaries, and veins provide the conduits for circulation. The cell membrane of cardiomyocytes and vascular smooth muscle cells are replete with ion channels that link action potentials to calcium transients from outside and within the cell that facilitate dynamic “sliding” of myofibrils to induce cellular contraction and relaxation. The initiation of action potentials occurs spontaneously in “nodal centers” in the heart but their coordinated pace is guided by signals from the autonomic nervous system for which there are receptors on cardiomyocytes and smooth muscle cells. Mitochondrial ATP provides the fuel for ion movements in, out and within the cell as well as for contraction. Fatty acids and oxygen are primary fuel substrates but other substrates can also be used though with less efficiency. Endothelial cells that line large and small blood vessels are key mediators of trans- or intercellular transport of nutrients, cellular waste, fuel substrates, proinflammatory cytokines, paracrine signalling (e.g., nitric oxide and endothelin as vasoactive molecules), and mediators of coagulation. Toxicity is usually a result of perturbing those usual molecular and mechanistic mediators.

Perturbations or altered function of these molecular mediators of usual CV physiology could be considered “failure modes” that can lead to a discrete number of tissue or organ-level dysfunctions or structural injuries. As an example, tissue and organ-level failures can be represented as an overt decrease in myocardial contractility, altered rhythmicity, a change in vasoactivity (vasoconstriction or vasodilation), loss of valve function, degeneration or death of the cellular constituents of the heart or blood vessels, an increase in coagulation or increase in inflammatory signaling (Table 1). The CV system, like many vital organ systems, also dynamically adapts to changes in functional demand to ensure survival of the organism and has the potential to compensate for a partial loss of function (i.e., a reserve capacity). Loss of that ability to respond to changes in demand or loss of reserve capacity is a form of injury or toxicity but more challenging to recognize in usual animal-based assessments and even human patients.

**TABLE 1:** Important cardiovascular failure modes.

Important cardiovascular failure modes	
Vasoactivity	Change in vascular tone (vasoconstriction or vasodilation)
Contractility	Change in contractile function (usually decreased in failure)
Rhythmicity	Altered rhythm/electrophysiology
Myocardial injury	Injury or death of cellular components of the myocardium (usually cardiomyocytes); infiltration or remodeling of myocardium
Endothelial injury	Injury or death of endothelial cells; change in coagulation; increased inflammatory signaling
Vascular injury	Injury or death of cellular constituents of blood vessels in any organ including the heart (most often arteries)
Valvulopathy	Valvular insufficiency (usually due to structural deformity or inflammation)

“Note. There are a finite number of ways that the cardiovascular system “fails” in disease or toxicity. These “failure modes” represent the focus of modeling systems that could detect drug bioactivities that might lead to or induce these failures.”

The “failure modes” that can lead to the discrete number of organ-level failures noted above can be quantitatively modeled using available systems. Those “modes” are represented as a perturbation of one or more of the key molecular pathways that support normal physiological function–many of which are evaluated in secondary pharmacology screens. They could be represented as a discrete set of “adverse outcome pathways” for which there is a growing database. Identifying these key failure modes and their associated adverse outcome pathways provides a framework for designing a relevant modeling paradigm that can be applied more broadly across the drug discovery and development process.

### 2.2 Leveraging technical capabilities

The ability to model fundamental elements of cellular biology at medium and high-throughput has expanded significantly over the past few decades. Individual receptors, transporters, enzymes and other mechanistic mediators of cell biology can be expressed in either native or artificial systems. Competitive and non-competitive (with natural substrates) binding and functional effects of endogenous or exogenous (e.g., drugs) molecules can be quantitatively evaluated.

Cell-based modeling systems of moderate throughput have the potential to provide insights into the link between those molecular bioactivities and alterations in normal cell function. A growth in more complex or three-dimensional modeling systems with tissue-level histoarchitecture offers the ability to link cellular function to more organ-level function increasing confidence in the *in vivo* relevance of the bioactivity without actually modeling the outcomes in an animal study. However, the value of these types of systems in the larger development picture is not yet established and more is needed to understand whether the time and expense are worthwhile.

**FIGURE 2:**
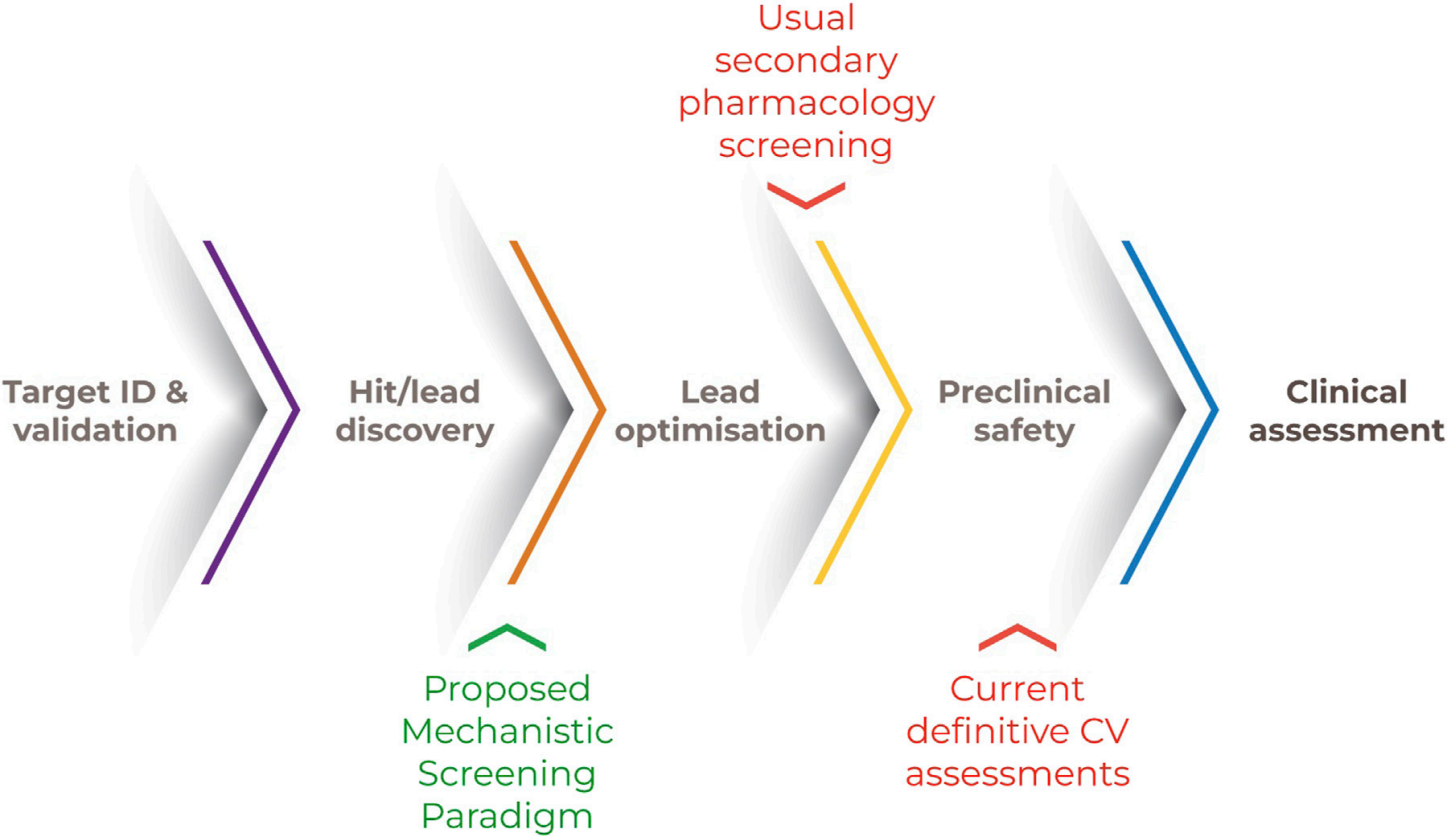
Tiered approach for human CV safety assessment. Note. A human-relevant and mechanistically-based screening strategy could include an initial screen for bioactivities of CV concern. The outcomes of subsequent and qualifying *in vitro* systems within a framework of recognized CV “failure modes” could be computationally extrapolated to human-relevant exposures. Actionable decisions include approaches to molecular design, a more informed candidate selection and bespoke clinical trial design.

This combination of biological understanding, experience in assessing CV disease and toxicity and rapid advances in technical capability provide a unique opportunity to define a novel, more broadly applicable and mechanistically informative approach to human CV safety assessment. A tiered approach is represented in Figure 2 along with the scientific justification, feasibility, and steps needed to advance the implementation of each tier.

### 2.3 Tier one: bioactivity/secondary pharmacology screening

These initial screens are targeted to identify important molecular interactions known to mediate toxic CV injury. The purpose of these screens is to provide initial high-throughput filters to identify bioactivities that relate to structure-activity and off-target effects. Results from these early screens constitute likely liabilities to be specifically evaluated in Tier Two and Three assessments.

Mechanistic or molecular targets are often included in secondary pharmacology screens already used by many drug developers to identify potential structural injuries or functional effects from unintended and off-target biological activities of a candidate drug prior to animal safety studies. For example, Bowes, et al. (2012), describes 44 molecular targets for secondary pharmacology screening including G-protein coupled receptors, nuclear receptors, enzymes, ion channels and transporters that can be evaluated in a combination of binding and functional assays (Bowes et al., 2012). This list represents the collective experience of a group of four major pharmaceutical companies who support their lead selection processes with routine “off-target” drug bioactivity screening to identify unexpected safety liabilities. Thirty of those targets have relevance to the CV system supported either by recognized CV-relevant biology (e.g., β-adrenergic receptors; voltage-gated calcium, sodium and potassium channels; histamine receptors) or previous association with adverse CV effects (e.g., 5-HT receptors, phosphodiesterase activity, cyclooxygenase activity).

Historically, bioactivity assessment at these targets has intended to support selective progression of lead compounds with the least liabilities, to tailor subsequent and more resource-intensive *in vivo* testing and to provide mechanistic insights into the effects seen in those *in vivo* systems. Activity at these CV-relevant targets often generates a suspicion of potential liability but often lacks verification until *after* a subsequent *in vivo* study is done and the expected change in function or structure is observed. Thus, the actionable impact or influence of those assessments in decision-making has been both delayed and dependent on subsequent investigation in an *in vivo* study. The actionable interpretation of these secondary pharmacology screens can also be confounded by the fact that the biological targets screened in these studies are often human-derived but the results are confirmed in *in vivo* animal studies which provides the potential for discordance in outcomes. Animal study outcomes are generally considered more actionable than the mechanistic *in vitro* activity modeled in the screen. Improving confidence in the *in vitro* screening assays would result in better decisions at this earlier stage and avoid inappropriately eliminating drugs due to results that have no bearing on clinical outcomes. Further, smaller drug development companies without a budget to run secondary pharmacology and *in vivo* studies, would have additional benefit of *in vitro* screening assays.

Current approaches to secondary pharmacology or bioactivity screening are primarily focused on mediators of functional changes in the CV system. Examples of this are β_1_-adrenergic receptor stimulation which could be expected to increase heart rate and force of contraction, adenosine receptors α2A activity where stimulatory binding might be expected to decrease blood pressure, and blocking or antagonism of the hERG potassium channel that would prolong the ECG QT interval providing an opportunity for arrhythmia. Paradoxically, some of the functional perturbations that might result from these activities could also produce structural injuries (Hanton, 2007). Potent stimulation of the β-adrenergic system can result in myocardial necrosis due to an imbalance produced by the increase in workload of a heart that is beating faster and harder relative to its perfusion which is decreased due to shortening of the relaxation/perfusion phase of the cardiac cycle (i.e., produces a “relative” ischemia). The astute and experienced cardiovascular pathobiologist would not need to model myocardial necrosis to recognize that it might occur, but a quantitative understanding of the cellular or tissue response to the β-adrenergic stimulation would build confidence that it is likely. Binding and even cell-based functional assays could provide that understanding.

Aside from the extrinsic signaling and bioactivities represented by a traditional secondary pharmacology or bioactivity screen, there are also key intracellular organellar targets of interest in the cellular constituents of the CV system as well as the potential for indiscriminate cell death (though the CV system might not be a primary target for indiscriminate cytotoxicity). Organelles of particular interest include mitochondria, the sarcoplasmic reticulum, nuclear and mitochondrial DNA, and lysosomes. The mitochondria are pre-eminent among those organelles given the profound energetic demands of a dynamically contractile organ like the heart and even the arterial vascular system. Several well-studied cardiotoxic agents have known mitochondrial effects including doxorubicin and the HIV therapeutic azidothymidine (Sardão et al., 2008; Varga et al., 2015; Wallace et al., 2020). Doxorubicin is also recognized to induce endoplasmic or sarcoplasmic reticulum (SR) stress presenting morphologically as dilation of the SR in experimental models (Ferrans, 1978; Bellini and Solcia, 1985; Iwasaki and Suzuki, 1991). Azidothymidine and doxorubicin both have recognized effects on DNA replication and/or repair. Though the heart is not a common target organ for the induction of drug-induced phospholipidosis, the antimalarial drug chloroquine which is also used in patients with lupus and rheumatoid arthritis is an example of a drug that does (Roos et al., 2002; Tönnesmann et al., 2013; Blignaut et al., 2019). Drug-induced phospholipidosis manifests as lysosomal accumulation of membranous whorls of phospholipids presumed to result from inhibition of phospholipase A2 degradation. Many maladaptive organellar injuries induce the common final injury pathway of apoptosis.

This set of intrinsic organellar bioactivities represents primary mediators of “cell health” in cardiomyocytes as well as most other cells in the body. A useful and holistic mechanistic screen could include a primary assessment of cell health in cardiomyocytes.

The biological responses to these mechanistic activities are well recognized and define hypotheses that could be the focus of further confirmatory *in vitro* assessment with human-derived cellular systems. An example of this is agonist activity on a β-adrenergic receptor. Depending on the selectivity for a specific receptor subtype (e.g., β_1_ vs. β_2_), a β-adrenergic agonist might be expected to increase the rate and force of cardiomyocyte contraction as well as relax arterial smooth muscle cells. Alternatively, a compound that blocks calcium channels could be expected to decrease the force of contraction of a cardiomyocyte and decrease vascular tone in arterial smooth muscle cells.

In addition to having some experiential confidence in the cellular targets of CV toxicity and their mechanistic mediators, we also have fair experience in characterizing response to injury in those targets. Cardiomyocytes respond to insult or injury with rhythmic or contractile dysfunction, changes in morphology (e.g., atrophy, hypertrophy, lipid accumulation) or cell death. Endothelial cells might exhibit a change in functional secretion of the vasoactive peptides nitric oxide or endothelin, may increase their expression of cell surface inflammatory signaling molecules, may alter their replication rate or undergo some form of cell death. Similarly, valvular stromal cells may proliferate or increase their production of matrix as they have been demonstrated to do with ALK inhibitors or 5-HT serotonin receptor agonists (Walker et al., 2004; Oyama et al., 2020). Vascular smooth muscle cells can increase or decrease their contractile tone. Extremes of that change in dynamic function can be accompanied by arterial smooth muscle injury *in vivo*. These changes in function and/or morphology are identifiable and even quantifiable in relevant *in vitro* systems.

#### 2.3.1 Feasibility and implementation of Tier I

Many of the biological targets represented in Tier I and the assays to measure them are well established, accessible, and scalable. The mechanistic secondary pharmacology screening already done routinely to identify important off-target bioactivities in most established pharmaceutical organizations can serve as a foundation for the proposed CV-specific Tier 1 screen (Bowes et al., 2012). The endpoints in these screens are already biased toward CV and neurologic bioactivities thus this well-established practice provides relevant substrate on which to build. It is likely that the existing portfolio of screening targets is incomplete. The breadth of a typical CV secondary pharmacology screen might be expanded to include additional mechanistic targets by systematically reviewing published experiences of cardiovascular toxicity and their associated pathogeneses leveraging our experiences further to identify novel targets. The xenobiotic effects identified in Tier I represent hypotheses to investigate in Tier II.

### 2.4 Tier II: *in vitro* failure mode evaluation

This tier further characterizes the targeted bioactivities identified in Tier I by evaluating those bioactivities in more complex and lower throughput *in vitro* cell and tissue-based confirmatory modeling systems. Tier II is designed to link those bioactivities to the ways that the CV system can “fail” at the organ level. Modeling failure modes unique to the CV system may provide more insight and confidence in potential impacts to the structural and functional changes in the CV system that are traditionally assessed in animal and clinical studies.

The Tier II evaluation assumes a finite set of well-recognized and tissue/organ-specific responses to injury (i.e., failures) as the basis for a framework that encompasses most of the ways that drug-induced CV toxicity will manifest. The cellular targets of these assessments are well characterized-cardiomyocytes, macro- and microvascular endothelial cells, valvular stromal cells and arterial smooth muscle cells (Berridge et al., 2016). More specifically, the cellular functions and biological pathways evaluated in Tier 1 are recognized to be targets of drug activity and mediators of toxicity at the cellular and tissue level that lead to the organ-level failure modes described above. For example, binding and inhibition of the L-type calcium channel could decrease cardiomyocyte contraction which would manifest *in vivo* as a decrease in ejection fraction or overt heart failure as represented in a relevant adverse outcome pathway (Margiotta-Casaluci, 2023). Many of these links are represented in a growing number of defined adverse outcomes pathways.

#### 2.4.1 Feasibility and implementation of Tier II

Rapid development of *in vitro* modeling systems that incorporate human induced pluripotent stem cells (iPSC)-derived cardiomyocytes, smooth muscle and endothelial cells offer opportunities to assess Tier 1 bioactivities for their cell, tissue or organ effects (i.e., Tier II assessment) (Dick et al., 2010; Rana et al., 2012; Doherty et al., 2013; Pointon et al., 2013; Grimm et al., 2018; Gintant et al., 2019). Some of these systems are rapidly becoming more physiologically-relevant incorporating important elements of 3D architecture, multiple cell types, contractile function, rhythmicity and microfluidic shear (Ogunrinade et al., 2002; Fernandez et al., 2016; Huebsch et al., 2016; Truskey, 2016; Atchison et al., 2017; Hoang et al., 2018; Zhang et al., 2020). An expanding portfolio of endpoints like contractile rate, action potential generation, rhythm and force; ATP generation, markers of oxidative stress, smooth muscle contraction or relaxation; as well as cell morphology and viability provide direct lines of sight to recognized responses to cardiac or vascular injury. Depending on the context of use, each of these systems will need to be characterized for its biological relevance, its toxicologic relevance, its analytical reproducibility and technical tractability according to pre-defined criteria. The stringency of these criteria will vary depending on the intended context of use of this approach (e.g., internal product design decision-making versus regulatory submissions). One of the next steps involves identifying these *in vitro* systems that are high-throughput and scalable for further characterization and incorporation into this proposed paradigm. Usual purveyors of *in vivo* animal-based modeling systems are providing valuable guidance about biological analytical performance standards to consider for *in vitro* and computational modeling developers (Fabre et al., 2020).

### 2.5 Tier III: concentration and IVIVE

In addition to the biological complexities above, this novel approach will require adequate incorporation of pharmacokinetic or toxicokinetic considerations. All the influences of absorption, distribution, metabolism, excretion, dose and time are at play *in vivo* and should be considered in extrapolating *in vitro* pharmacobiology to *in vivo* outcomes for CV risk assessment. These challenges are not unique to CV, however, and are increasingly managed with *in vitro* screening or modeling approaches today that include complementary modeling of those fundamental activities (absorption assays, hepatic metabolism and clearance, physiologically based pharmacokinetic [PBPK] computation, etc.) (Jones et al., 2015; Sager et al., 2015; Zhuang and Lu, 2016). Like receptor affinity studies, these assessments are generally done in humanized models and thus enhance the human-relevance of this screening strategy.

In Tier 3, the outcomes of the “failure mode” assessment above will be contextualized against likely PK and human-relevant exposure considerations via *in vitro* to *in vivo* extrapolation (IVIVE) and other computational modeling approaches (Casey et al., 2018; Chang et al., 2022). This modeling and measuring of concentration-dependent human and *in vivo*-relevant CV bioactivity will subsequently provide a substrate for extrapolation to patients-an exercise known as IVIVE. The purpose of this step is to enable *in vivo* extrapolation and determination of the likelihood of patient risk for the biological or toxicological activity defined.

#### 2.5.1 Tier III feasibility and implementation

Human pharmacokinetic and even toxicokinetic modeling are routine. Animal plasma exposures (AUC, C_max_, free and bound fractions) are integrated with *in vitro* assessments of human plasma protein binding and intrinsic clearance assessments in human primary liver cells to predict a likely human plasma exposure. Computational approaches to extrapolating *in vitro* concentrations to human plasma exposures that would be required to produce a specific bioactivity have also been developed and could be adapted to this screening paradigm (Bell et al., 2018; Wambaugh et al., 2018; Honda et al., 2019).

### 2.6 Tier IV: actionable decisions about a candidate compound

Tier IV involves the integration of the cumulative evidence derived from the first 3 assessment tiers with the aim of supporting decisions around molecular design, candidate selection, *in vivo* follow-up testing, and/or bespoke clinical trial design. This will likely require a novel decision framework that is more specific and focused than the fairly high-level observational decision framework applied to the usual *in vivo* animal-based system. It will also require understanding and confidence in data that is different than that usually used in safety assessments. As that confidence grows, Tier IV could evolve over time allowing for less or no reliance on *in vivo* data and support earlier decision-making.

Tier I uses a broad set of specific (receptors, ion channels, enzymes) and non-specific (e.g., cell health assessments) biological assessments to generate hypotheses that a CV liability is a potential concern for a novel drug candidate. Tier II assessments qualify those bioactivies for their CV-specific relevance in more complex modeling systems with the ability to model normal and abnormal or pathologic biology at the tissue and organ level. Tier III contextualizes potential liabilities with the concentration or dose at which those liabilities are likely to occur.

Avila et al. (2020) articulated a short series of high-level questions generally considered in human drug safety assessment. Within those questions are a myriad of potential considerations that relate to all the major organ systems of human biology. We contend that our understanding of human responses to injury or disease and our history of characterizing drug-induced toxicity positions a global community of toxicologists to define a discrete set of target-organ based questions that could guide a novel and more mechanistic approach to drug safety assessment. This novel decision framework is a key enabler to adopting a novel paradigm.

#### 2.6.1 Tier IV feasibility and implementation

Confidence in the results of Tiers I-III are critical to the success of this last tier to avoid duplicating efforts in the *in vivo* studies (as discussed below). Once confidence has been established, Tier III results can be used to inform molecule design and an integrated risk assessment. Focusing the risk assessment on the predefined bioactivities will be necessary to best characterize the liabilities that may exist. Results can also be used to inform clinical trial design such that the suspected liabilities are monitored (e.g., ECG screening in early stage 1 trials for suspected proarrhythmic risk).

## 3 Implementing the tiered approach

The four-tiered approach described above aims to leverage our significant knowledge and experience with characterizing CV responses to xenobiotic-induced injury or dysfunction. It intends to expand the use of existing capabilities for detecting and measuring biological events at the cellular level in a framework that is more mechanistic, evidence-based and applicable across the drug discovery and development continuum. To be decision-enabling, it will need to be as complete as our knowledge allows, be human-relevant, be toxicologically-relevant and have the confidence of the decision makers who will use the outcomes of these assessments. That confidence will come from demonstrating the human biological relevance of the modeling systems, the CV relevance of the endpoints that are measured and the line of sight from *in vitro* mechanistic perturbations and changes in cell function to organ system-level clinical outcomes.

As noted above, our experience with secondary pharmacology screening and basic cell health assessments is a strong basis for our confidence in those assessments (i.e., we have experience using them). The biological scope of those assessments should likely be expanded to ensure that all CV-relevant molecular targets are represented (i.e., that the assessment is sufficiently holistic) and, though assays for most of the endpoints of interest are commercially available, they would benefit from being integrated into a discrete portfolio of assays amenable to high-throughput application to be useful in early discovery efforts.

Human cell-based modeling capabilities in simple and more complex configurations (e.g., 3D, multicellular) are progressing rapidly increasing confidence in both their human and *in vivo* relevance. Demonstrating that relevance is an important contributor to confidence-building. For example, a cardiomyocyte should rhythmically beat in response to an “electrical stimulation,” express troponins as components of its contractile apparatus, use fatty acids as a preferential fuel source and cycle calcium in and out of intra- and extracellular stores. There are a growing number of publications providing guidance on *in vivo* relevant biological features that should be modeled in *in vitro* systems (Guth et al., 2019; Ribeiro et al., 2019).

Also, cellular constituents of the CV system should replicate responses to known CV-active pharmacologic and toxic agents. Pharmacologic agents (i.e., drugs), in particular, offer the opportunity to benchmark bioactivities in *in vitro* systems against human responses exploring the dynamic range of bioactivity using cardio-active drugs with known pharmacologic mechanisms of action as tools to qualify drug responses. “Validating” the systems analytically will establish their reproducibility. Confidence in the outcomes of the effort will be supported by working the paradigm with a blinded panel of compounds with known human activity to ensure appropriate translation. These compounds must include drugs with known mechanisms that elicit toxicity via recognized and CV-relevant pathways rather than cardiotoxic drugs with known outcomes but unknown mechanisms. Real-time integration of this paradigm with contemporary approaches will fill an existing gap in our current approaches (i.e., identify true development-limiting liabilities earlier in development).

## 4 Limitations

This paradigm is not offered without recognizing that the cardiovascular system is physiologically integrated, has significant interdependencies among its individual tissue and organ components, and a number of controlling influences from non-CV organ systems. We propose that you do not have to replicate the entire organism to predict what would happen at the organ system level. In order to leverage the proposed higher throughput and human-relevant, albeit more reductionist approach, we will need to apply principles of systems biology by additively or computationally integrating outcomes and extrapolating them to the *in vivo* human context. *In vitro* assessment of phosphodiesterase activity in a secondary pharmacology screen is an example. Inhibition of phosphodiesterase activity in an *in vitro* assay would be expected to induce vasorelaxation in a cell-based *in vitro* assessment of dynamic vascular smooth muscle activity. Our understanding of vascular physiology provides confidence in the prediction that, *in vivo*, blood pressure would drop and heart rate would increase since these are usual physiologic responses to that bioactivity (Losco et al., 2004; Hanton et al., 2008; Zhang et al., 2008). Likewise, a drug candidate that increased the force of contraction in a dynamically contracting cardiomyocyte system would be expected to increase pulse pressure *in vivo* (Banfor et al., 2008; Abram et al., 2015). Alternatively, cardiomyocyte cytotoxicity and death *in vitro* would be expected to exhibit myocardial necrosis and increased circulating cTn *in vivo*. While current animal studies reveal changes that are clinically undetectable, similarly cell-based assays may reveal bioactivity that never manifests in patients. An important element of this more mechanistic approach requires some bridging and insight into the quantitative translation of molecular bioactivity to *in vivo* outcomes. Those insights could come from benchmarking against historical experiences of that translation leveraging marketed drugs with these intentional pharmacological activities.

A potential obstacle to the adoption of this, or any other more reductionist and mechanistic approach to safety assessment, is the concern for liabilities that might not be modeled in a more reductionist system (i.e., the fear of the unknown). Likewise, novel therapeutic modalities like stem cell and gene therapies will require re-thinking our current paradigm but benefit even more from species-relevant modeling systems. Interestingly, “unknowns” occur with the current animal-based approach in the form of unexpected and, occasionally, severe liabilities occurring in patients that were not recognized preclinically (Forrest et al., 2008; Harirforoosh et al., 2014; Wojcik et al., 2015). As the current testing paradigm has limited capabilities to be reflexively modified based on outcomes in the clinical setting, this more mechanistic and hypothesis-based paradigm could be modulated over time to be increasingly more predictive based upon clinical outcomes data.

Aside from the challenges above and despite the seemingly inherent value of a human-based modeling approach, the significant historical weight of our experience using animal data to make human decisions suggests that animal studies are likely to remain fundamental in pharmaceutical safety assessment for the foreseeable future (Avila et al., 2020). The rapid proliferation of human-relevant cell-based modeling systems has already prompted broader use of these systems for both regulatory and non-regulatory decision-making. Yang et al. (2022) recently reported a significant increase in the number of regulatory submissions to the FDA that included safety assessment data derived from human iPSC cardiomyocytes for both proarrhythmic and non-proarrhythmic safety liabilities using more mechanistic endpoints than usual *in vivo* animal studies. Though those data were submitted as complements to a usual safety assessment package, the potential exists for their use as alternatives to traditional data as confidence grows in their usefulness and relevance to human outcomes.

We recognize that the retention of animal-based CV risk assessment methods in parallel with the implementation of a novel, mechanistic-based paradigm may cause friction. What happens when a compound is incorrectly predicted to pose human CV safety liabilities in animal studies but not in a human-derived *in vitro* system? Or *vice versa*? If one assumes that animal studies have been reasonable surrogates for human outcomes and that the new approach is equally or more translational, then these disparities should be uncommon. If they are not, we may reveal a significant weakness in that assumption or even challenge our belief that *in vivo* outcomes can be reasonably modeled in a more reductionist *in vitro* system. Whatever the outcome, the proposed new paradigm will benefit from a prospective rigorous and evidence-based validation to assess the strengths and limitations of its application. These assessments will undoubtedly bring significant value by recognizing human CV liabilities earlier in development and may prompt us to improve our ability to translate preclinical modeling to clinical outcomes–an improvement sorely needed in drug development.

## 5 Conclusion

**FIGURE 3:**
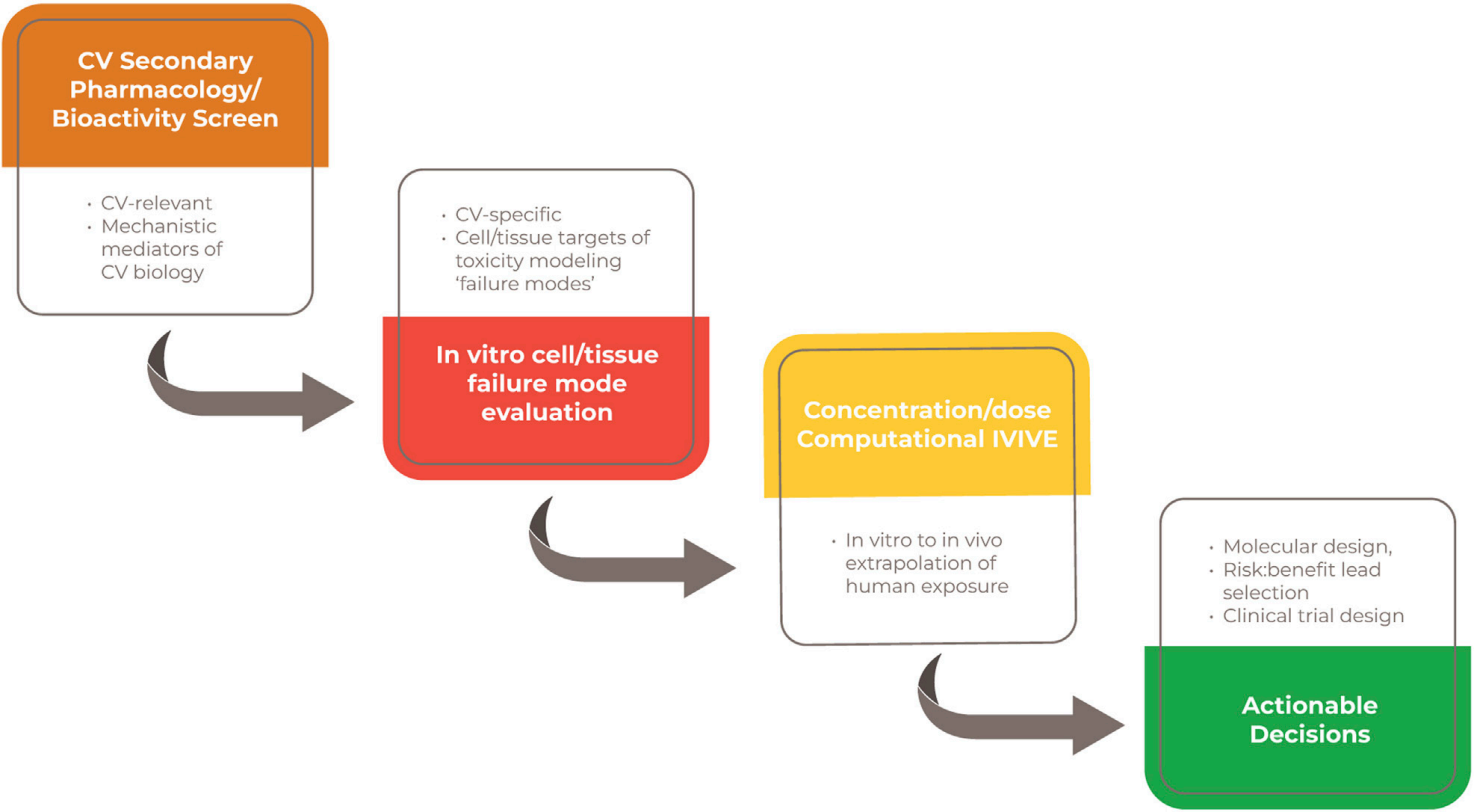
Value propositions for an earlier integration of a human-relevant and mechanistic screening strategy.

Animal study-based safety testing of novel drug candidates prior to clinical testing has prevented unintended adverse effects in most patients. Their application late in preclinical development and the potential that they under- or over-estimate patient risks likely represent important contributions to high attrition and cost of drug development. This is particularly true for CV safety liabilities. A higher throughput, more human-relevant and mechanistically informative, objectively evaluated screening strategy has the opportunity to mitigate that contribution. A human-relevant and higher throughput paradigm could identify true human liabilities earlier in development, support a more informed risk/benefit lead selection and guide the design of subsequent preclinical and clinical *in vivo* studies (Figure 3). The multidisciplinary and cross-sector HESI Cardiac Safety Technical Committee is taking on the challenge of defining and testing this strategy leveraging our understanding of the CV system, our experiences modeling toxicity in that system and the substantial proliferation of modeling platforms that would support such an approach.

This HESI Committee is a multidisciplinary team of *in vivo* and *in vitro* scientists with expertise in cardiovascular toxicobiology, cell-based modeling and computational approaches representing the pharmaceutical industry, government, academia and developers of the kinds of systems that will be needed to support this strategy (https://hesiglobal.org/cardiac-safety/). This group has been collaborating to align on the fundamental biological components (the “failure modes”) of the CV system that should be modeled to drive this proposed approach. In addition to identifying and understanding the failure modes, the Cardiac Safety Technical Committee is actively working to bridge the gaps between *in vitro*, *in vivo* data and clinical outcomes by conducting research and generating novel data. The responsibility of such a paradigm shift falls to everyone and HESI is ensuring collaboration across sectors, which will allow for an environment where change is possible. Working together will also combine expertise and experience to ensure new approaches are applied with the proper context of use and the appropriate stage of development.

HESI and similar consortia have published validation studies and best practices and continue to do so as part of their remit. Additional data along with case studies and training on data interpretation across pharmaceutical companies and regulatory agencies are still needed. A clear path for inclusion of such data in new drug submissions with buy in from all stakeholders is ultimately needed and can be achieved through further scientific development, regulatory acceptance and a tiered approach to prediction.

The magnitude of change proposed here is not to be taken lightly and it cannot be easily accomplished without full engagement of a committed biomedical community and substantial support and investment. A value proposition for a novel paradigm with the attributes described above is clear. Building the capability described will require that we leverage our collective experiences as well as technical advancements in *in vitro* and computational modeling. Gaining the confidence to make important decisions that ensure patient safety but also optimizes their opportunity for beneficial therapies will require a frameshift in a risk assessment culture that has been understandably conservative and incremental in its evolution and adoption of novel approaches. Bold change has inherent risks but we contend that we know enough to mitigate those risks to the ultimate benefit of patients and drug developers.

## References

[B1] Abram S. Arruda-Olson A. M. Scott C. G. Pellikka P. A. Nkomo V. T. Oh J. K. (2015). Typical blood pressure response during dobutamine stress echocardiography of patients without known cardiovascular disease who have normal stress echocardiograms. Eur. Heart J. Cardiovasc Imaging 17 (5), 557–563. 10.1093/ehjci/jev165 26206464 PMC6279206

[B2] Atchison L. Zhang H. Cao K. Truskey G. A. (2017). A tissue engineered blood vessel model of hutchinson-gilford progeria syndrome using human iPSC-derived smooth muscle cells. Sci. Rep. 7 (1), 8168. 10.1038/s41598-017-08632-4 28811655 PMC5557922

[B3] Avila A. M. Bebenek I. Bonzo J. A. Bourcier T. Davis Bruno K. L. Carlson D. B. (2020). An FDA/CDER perspective on nonclinical testing strategies: classical toxicology approaches and new approach methodologies (NAMs). Regul. Toxicol. Pharmacol. 114, 104662. 10.1016/j.yrtph.2020.104662 32325112

[B4] Banfor P. N. Preusser L. C. Campbell T. J. Marsh K. C. Polakowski J. S. Reinhart G. A. (2008). Comparative effects of levosimendan, OR-1896, OR-1855, dobutamine, and milrinone on vascular resistance, indexes of cardiac function, and O2 consumption in dogs. Am. J. Physiol. Heart Circ. Physiol. 294 (1), H238–H248. 10.1152/ajpheart.01181.2007 17982006

[B5] Bell S. M. Chang X. Wambaugh J. F. Allen D. G. Bartels M. Brouwer K. L. R. (2018). *In vitro* to *in vivo* extrapolation for high throughput prioritization and decision making. Toxicol Vitro 47, 213–227. 10.1016/j.tiv.2017.11.016 PMC7393693 29203341

[B6] Bellini O. Solcia E. (1985). Early and late sarcoplasmic reticulum changes in doxorubicin cardiomyopathy. An ultrastructural investigation with the zinc iodide-osmium tetroxide (ZIO) technique. Virchows Arch. B Cell Pathol. Incl. Mol. Pathol. 49 (1), 137–152. 10.1007/BF02912092 2866624

[B7] Berridge B. R. Hoffmann P. Turk J. R. Sellke F. Gintant G. Hirkaler G. (2013). Integrated and translational nonclinical *in vivo* cardiovascular risk assessment: gaps and opportunities. Regul. Toxicol. Pharmacol. 65 (1), 38–46. 10.1016/j.yrtph.2012.09.007 23044254

[B8] Berridge B. R. Mowat V. Nagai H. Nyska A. Okazaki Y. Clements P. J. (2016). Non-proliferative and proliferative lesions of the cardiovascular system of the rat and mouse. J. Toxicol. Pathol. 29 (3), 1S–47S. 10.1293/tox.29.3S-1 PMC5013710 27621537

[B9] Blignaut M. Espach Y. van Vuuren M. Dhanabalan K. Huisamen B. (2019). Revisiting the cardiotoxic effect of chloroquine. Cardiovasc Drugs Ther. 33 (1), 1–11. 10.1007/s10557-018-06847-9 30635818

[B10] Bowes J. Brown A. J. Hamon J. Jarolimek W. Sridhar A. Waldron G. (2012). Reducing safety-related drug attrition: the use of *in vitro* pharmacological profiling. Nat. Rev. Drug Discov. 11 (12), 909–922. 10.1038/nrd3845 23197038

[B11] Casey W. M. Chang X. Allen D. G. Ceger P. C. Choksi N. Y. Hsieh J.-H. (2018). Evaluation and optimization of pharmacokinetic models for *in vitro* to *in vivo* extrapolation of estrogenic activity for environmental chemicals. Environ. Health Perspect. 126 (9), 097001. 10.1289/EHP1655 30192161 PMC6375436

[B12] Chang X. Tan Y.-M. Allen D. G. Bell S. Brown P. C. Browning L. (2022). IVIVE: facilitating the use of *in vitro* toxicity data in risk assessment and decision making. Toxics 10 (5), 232. 10.3390/toxics10050232 35622645 PMC9143724

[B13] Dick E. Rajamohan D. Ronksley J. Denning C. (2010). Evaluating the utility of cardiomyocytes from human pluripotent stem cells for drug screening. Biochem. Soc. Trans. 38 (4), 1037–1045. 10.1042/BST0381037 20659000

[B14] Dogterom P. Zbinden G. Reznik G. K. (1992). Cardiotoxicity of vasodilators and positive inotropic/vasodilating drugs in dogs: an overview. Crit. Rev. Toxicol. 22 (3-4), 203–241. 10.3109/10408449209145324 1388706

[B15] Doherty K. R. Wappel R. L. Talbert D. R. Trusk P. B. Moran D. M. Kramer J. W. (2013). Multi-parameter *in vitro* toxicity testing of crizotinib, sunitinib, erlotinib, and nilotinib in human cardiomyocytes. Toxicol. Appl. Pharmacol. 272 (1), 245–255. 10.1016/j.taap.2013.04.027 23707608

[B16] Fabre K. Berridge B. Proctor W. R. Ralston S. Will Y. Baran S. W. (2020). Introduction to a manuscript series on the characterization and use of microphysiological systems (MPS) in pharmaceutical safety and ADME applications. Lab. Chip 20 (6), 1049–1057. 10.1039/c9lc01168d 32073020

[B17] Fernandez C. E. Yen R. W. Perez S. M. Bedell H. W. Povsic T. J. Reichert W. M. (2016). Human vascular microphysiological system for *in vitro* drug screening. Sci. Rep. 6 (1), 21579. 10.1038/srep21579 26888719 PMC4757887

[B18] Ferrans V. J. (1978). Overview of cardiac pathology in relation to anthracycline cardiotoxicity. Cancer Treat. Rep. 62 (6), 955–961. 352510

[B19] Ferri N. Siegl P. Corsini A. Herrmann J. Lerman A. Benghozi R. (2013). Drug attrition during pre-clinical and clinical development: understanding and managing drug-induced cardiotoxicity. Pharmacol. Ther. 138 (3), 470–484. 10.1016/j.pharmthera.2013.03.005 23507039

[B20] Forrest M. J. Bloomfield D. Briscoe R. J. Brown P. N. Cumiskey A.-M. Ehrhart J. (2008). Torcetrapib-induced blood pressure elevation is independent of CETP inhibition and is accompanied by increased circulating levels of aldosterone. Br. J. Pharmacol. 154 (7), 1465–1473. 10.1038/bjp.2008.229 18536749 PMC2440088

[B21] Gintant G. Burridge P. Gepstein L. Harding S. Herron T. Hong C. (2019). Use of human induced pluripotent stem cell–derived cardiomyocytes in preclinical cancer drug cardiotoxicity testing: a scientific statement from the American heart association. Circ. Res. 125 (10), e75–e92. 10.1161/RES.0000000000000291 31533542 PMC7398423

[B22] Grimm F. A. Blanchette A. House J. S. Ferguson K. Hsieh N. H. Dalaijamts C. (2018). A human population-based organotypic *in vitro* model for cardiotoxicity screening. ALTEX 35 (4), 441–452. 10.14573/altex.1805301 29999168 PMC6231908

[B23] Guth B. D. Engwall M. Eldridge S. Foley C. M. Guo L. Gintant G. (2019). Considerations for an *in vitro*, cell-based testing platform for detection of adverse drug-induced inotropic effects in early drug development. Part 1: general considerations for development of novel testing platforms. Front. Pharmacol. 10, 884. 10.3389/fphar.2019.00884 31447679 PMC6697071

[B24] Hanton G. (2007). Preclinical cardiac safety assessment of drugs. Drugs R. D. 8 (4), 213–228. 10.2165/00126839-200708040-00002 17596108

[B25] Hanton G. Sobry C. Daguès N. Provost J.-P. Le Net J.-L. Comby P. (2008). Characterisation of the vascular and inflammatory lesions induced by the PDE4 inhibitor CI-1044 in the dog. Toxicol. Lett. 179 (1), 15–22. 10.1016/j.toxlet.2008.03.009 18485625

[B26] Harirforoosh S. Asghar W. Jamali F. (2014). Adverse effects of nonsteroidal antiinflammatory drugs: an update of gastrointestinal, cardiovascular and renal complications. J. Pharm. Pharm. Sci. 16 (5), 821–847. 10.18433/j3vw2f 24393558

[B27] Haschek W. M. Rousseaux C. G. Wallig M. A. (2013). Haschek and Rousseaux's handbook of toxicologic pathology. Amsterdam, Netherlands: Academic Press.

[B28] Hoang P. Wang J. Conklin B. R. Healy K. E. Ma Z. (2018). Generation of spatial-patterned early-developing cardiac organoids using human pluripotent stem cells. Nat. Protoc. 13 (4), 723–737. 10.1038/nprot.2018.006 29543795 PMC6287283

[B29] Honda G. S. Pearce R. G. Pham L. L. Setzer R. W. Wetmore B. A. Sipes N. S. (2019). Using the concordance of *in vitro* and *in vivo* data to evaluate extrapolation assumptions. PLoS One 14 (5), e0217564. 10.1371/journal.pone.0217564 31136631 PMC6538186

[B30] Huebsch N. Loskill P. Deveshwar N. Spencer C. I. Judge L. M. Mandegar M. A. (2016). Miniaturized iPS-cell-derived cardiac muscles for physiologically relevant drug response analyses. Sci. Rep. 6 (1), 24726. 10.1038/srep24726 27095412 PMC4837370

[B31] ICH Harmonised Tripartite Guideline (2009). Guidance on nonclinical safety studies for the conduct of human clinical trials and marketing authorization for pharmaceuticals M3(R2). ICH, New York, NY, USA.

[B32] Iwasaki T. Suzuki T. (1991). Ultrastructural alterations of the myocardium induced by doxorubicin. A scanning electron microscopic study. Virchows Arch. B Cell Pathol. Incl. Mol. Pathol. 60 (1), 35–39. 10.1007/BF02899525 1673275

[B33] Jones H. Chen Y. Gibson C. Heimbach T. Parrott N. Peters S. (2015). Physiologically based pharmacokinetic modeling in drug discovery and development: a pharmaceutical industry perspective. Clin. Pharmacol. Ther. 97 (3), 247–262. 10.1002/cpt.37 25670209

[B34] Katz A. M. (2010). Physiology of the heart. Philadelphia, PA, USA: Wolters Kluwer Health/Lippincott Williams & Wilkins Health.

[B35] Kloner R. A. Goggin P. Goldstein I. Hackett G. Kirby M. G. Osterloh I. (2018). A new perspective on the nitrate-phosphodiesterase type 5 inhibitor interaction. J. Cardiovasc Pharmacol. Ther. 23 (5), 375–386. 10.1177/1074248418771896 29739235

[B36] Laverty H. Benson C. Cartwright E. Cross M. Garland C. Hammond T. (2011). How can we improve our understanding of cardiovascular safety liabilities to develop safer medicines? Br. J. Pharmacol. 163 (4), 675–693. 10.1111/j.1476-5381.2011.01255.x 21306581 PMC3111672

[B37] Losco P. E. Evans E. W. Barat S. A. Blackshear P. E. Reyderman L. Fine J. S. (2004). The toxicity of SCH 351591, a novel phosphodiesterase-4 inhibitor, in cynomolgus monkeys. Toxicol. Pathol. 32 (3), 295–308. 10.1080/01926230490431493 15204971

[B38] Margiotta-Casaluci L. (2023). L-type calcium channel blockade leading to heart failure via decrease in cardiac contractility. [Online]. Available: https://aopwiki.org/aops/261 (Accessed July 6, 2023).

[B39] Monticello T. M. Jones T. W. Dambach D. M. Potter D. M. Bolt M. W. Liu M. (2017). Current nonclinical testing paradigm enables safe entry to First-In-Human clinical trials: the IQ consortium nonclinical to clinical translational database. Toxicol. Appl. Pharmacol. 334, 100–109. 10.1016/j.taap.2017.09.006 28893587

[B40] Ogunrinade O. Kameya G. T. Truskey G. A. (2002). Effect of fluid shear stress on the permeability of the arterial endothelium. Ann. Biomed. Eng. 30 (4), 430–446. 10.1114/1.1467924 12085996

[B41] Oyama M. A. Elliott C. Loughran K. A. Kossar A. P. Castillero E. Levy R. J. (2020). Comparative pathology of human and canine myxomatous mitral valve degeneration: 5HT and TGF-β mechanisms. Cardiovasc Pathol. 46, 107196. 10.1016/j.carpath.2019.107196 32006823 PMC7078050

[B42] Pierson J. B. Berridge B. R. Brooks M. B. Dreher K. Koerner J. Schultze A. E. (2013). A public–private consortium advances cardiac safety evaluation: achievements of the HESI Cardiac Safety Technical Committee. J. Pharmacol. Toxicol. Methods 68 (1), 7–12. 10.1016/j.vascn.2013.03.008 23567075

[B43] Pointon A. Abi-Gerges N. Cross M. J. Sidaway J. E. (2013). Phenotypic profiling of structural cardiotoxins *in vitro* reveals dependency on multiple mechanisms of toxicity. Toxicol. Sci. 132 (2), 317–326. 10.1093/toxsci/kft005 23315586

[B44] Pugsley M. K. Towart R. Authier S. Gallacher D. J. Curtis M. J. (2010). Non-clinical models: validation, study design and statistical consideration in safety pharmacology. J. Pharmacol. Toxicol. Methods 62 (1), 1–3. 10.1016/j.vascn.2010.06.003 20601022

[B45] Rana P. Anson B. Engle S. Will Y. (2012). Characterization of human-induced pluripotent stem cell–derived cardiomyocytes: bioenergetics and utilization in safety screening. Toxicol. Sci. 130 (1), 117–131. 10.1093/toxsci/kfs233 22843568

[B46] Ribeiro A. J. S. Guth B. D. Engwall M. Eldridge S. Foley C. M. Guo L. (2019). Considerations for an *in vitro*, cell-based testing platform for detection of drug-induced inotropic effects in early drug development. Part 2: designing and fabricating microsystems for assaying cardiac contractility with physiological relevance using human iPSC-cardiomyocytes. Front. Pharmacol. 10, 934. 10.3389/fphar.2019.00934 31555128 PMC6727630

[B47] Roos J. M. Aubry M.-C. Edwards W. D. (2002). Chloroquine cardiotoxicity: clinicopathologic features in three patients and comparison with three patients with Fabry disease. Cardiovasc Pathol. 11 (5), 277–283. 10.1016/s1054-8807(02)00118-7 12361838

[B48] Sager J. E. Yu J. Ragueneau-Majlessi I. Isoherranen N. (2015). Physiologically based pharmacokinetic (PBPK) modeling and simulation approaches: a systematic review of published models, applications, and model verification. Drug Metab. Dispos. 43 (11), 1823–1837. 10.1124/dmd.115.065920 26296709 PMC4613950

[B49] Sardão V. A. Pereira S. L. Oliveira P. J. (2008). Drug-induced mitochondrial dysfunction in cardiac and skeletal muscle injury. Expert Opin. Drug Saf. 7 (2), 129–146. 10.1517/14740338.7.2.129 18324876

[B50] Schurtz G. Mewton N. Lemesle G. Delmas C. Levy B. Puymirat E. (2023). Beta-blocker management in patients admitted for acute heart failure and reduced ejection fraction: a review and expert consensus opinion. Front. Cardiovasc Med. 16 (10), 1263482. 10.3389/fcvm.2023.1263482 PMC10693984 38050613

[B51] Sirenko O. Grimm F. A. Ryan K. R. Iwata Y. Chiu W. A. Parham F. (2017). *In vitro* cardiotoxicity assessment of environmental chemicals using an organotypic human induced pluripotent stem cell-derived model. Toxicol. Appl. Pharmacol. 322, 60–74. 10.1016/j.taap.2017.02.020 28259702 PMC5734940

[B52] Stucki A. O. Barton-Maclaren T. S. Bhuller Y. Henriquez J. E. Henry T. R. Hirn C. (2022). Use of new approach methodologies (NAMs) to meet regulatory requirements for the assessment of industrial chemicals and pesticides for effects on human health. Front. Toxicol. 4, 964553. 10.3389/ftox.2022.964553 36119357 PMC9475191

[B53] Tönnesmann E. Kandolf R. Lewalter T. (2013). Chloroquine cardiomyopathy – a review of the literature. Immunopharmacol. Immunotoxicol. 35 (3), 434–442. 10.3109/08923973.2013.780078 23635029

[B54] Truskey G. (2016). Advancing cardiovascular tissue engineering [version 1; peer review: 3 approved]. F1000Res 5 (1045), 1045. 10.12688/f1000research.8237.1 PMC4890312 27303643

[B55] Varga Z. V. Ferdinandy P. Liaudet L. Pacher P. (2015). Drug-induced mitochondrial dysfunction and cardiotoxicity. Am. J. Physiol. Heart Circ. Physiol. 309 (9), H1453–H1467. 10.1152/ajpheart.00554.2015 26386112 PMC4666974

[B56] Walker G. A. Masters K. S. Shah D. N. Anseth K. S. Leinwand L. A. (2004). Valvular myofibroblast activation by transforming growth factor-beta: implications for pathological extracellular matrix remodeling in heart valve disease. Circ. Res. 95 (3), 253–260. 10.1161/01.RES.0000136520.07995.aa 15217906

[B57] Wallace K. B. Sardão V. A. Oliveira P. J. (2020). Mitochondrial determinants of doxorubicin-induced cardiomyopathy. Circ. Res. 126 (7), 926–941. 10.1161/CIRCRESAHA.119.314681 32213135 PMC7121924

[B58] Wambaugh J. F. Hughes M. F. Ring C. L. MacMillan D. K. Ford J. Fennell T. R. (2018). Evaluating *in vitro*-*in vivo* extrapolation of toxicokinetics. Toxicol. Sci. 163 (1), 152–169. 10.1093/toxsci/kfy020 29385628 PMC5920326

[B59] Waring M. J. Arrowsmith J. Leach A. R. Leeson P. D. Mandrell S. Owen R. M. (2015). An analysis of the attrition of drug candidates from four major pharmaceutical companies. Nat. Rev. Drug Discov. 14 (7), 475–486. 10.1038/nrd4609 26091267

[B60] Whitebread S. Dumotier B. Armstrong D. Fekete A. Chen S. Hartmann A. (2016). Secondary pharmacology: screening and interpretation of off-target activities – focus on translation. Drug Discov. Today 21 (8), 1232–1242. 10.1016/j.drudis.2016.04.021 27140035

[B61] Wojcik T. Szczesny E. Chlopicki S. (2015). Detrimental effects of chemotherapeutics and other drugs on the endothelium: a call for endothelial toxicity profiling. Pharmacol. Rep. 67 (4), 811–817. 10.1016/j.pharep.2015.03.022 26321285

[B62] Yang X. Ribeiro A. J. S. Pang L. Strauss D. G. (2022). Use of human iPSC-CMs in nonclinical regulatory studies for cardiac safety assessment. Toxicol. Sci. 190 (2), 117–126. 10.1093/toxsci/kfac095 36099065

[B63] Zhang J. Snyder R. D. Herman E. H. Knapton A. Honchel R. Miller T. (2008). Histopathology of vascular injury in sprague-dawley rats treated with phosphodiesterase IV inhibitor SCH 351591 or SCH 534385. Toxicol. Pathol. 36 (6), 827–839. 10.1177/0192623308322308 18776163

[B64] Zhang Q. Zhang X. Truskey G. A. (2020). Vascular microphysiological systems to model diseases. Cell Gene Ther. Insights 6 (1), 93–102. 10.18609/cgti.2020.012 32431950 PMC7236815

[B65] Zhuang X. Lu C. (2016). PBPK modeling and simulation in drug research and development. Acta Pharm. Sin. B 6 (5), 430–440. 10.1016/j.apsb.2016.04.004 27909650 PMC5125732

